# Supplementary material for: P-TRAP: a Panicle Trait Phenotyping tool
Source: BMC Plant Biol. 2013 Aug 29;13:122. doi: 10.1186/1471-2229-13-122 (PMC3848748; doi:10.1186/1471-2229-13-122)
Supplement: Additional file 7 — P-TRAP source code. The Netbeans project that contains the source code of the application. [file 1471-2229-13-122-S7.zip › javadoc.zip!/javadoc/org-bioalg-riceprojecttype/constant-values.html]

Constant Field Values (RiceProjectType)


JavaScript is disabled on your browser.


- Package
- Class
- Tree
- Deprecated
- Index
- Help

*org.bioalg.riceprojecttype 1.0*

- Prev
- Next

- Frames
- No Frames

- All Classes

# Constant Field Values

## Contents

- org.bioalg.\*

## org.bioalg.\*

- org.bioalg.riceprojecttype.RiceProjectFactory

  | Modifier and Type | Constant Field | Value |
  | --- | --- | --- |
  |  |  |  |
  | --- | --- | --- |
  | `public static final java.lang.String` | `IMAGE_DIR` | `"images"` |
  | `public static final java.lang.String` | `PARTICLES_RESULT_DIR` | `"Particles"` |
  | `public static final java.lang.String` | `PARTICLES_RESULT_FILE` | `"GrainsTraits.csv"` |
  | `public static final java.lang.String` | `PROJECT_DIR` | `"riceproj"` |
  | `public static final java.lang.String` | `RESULTS_DETAILS_FILE` | `"AllTraits.csv"` |
  | `public static final java.lang.String` | `RESULTS_DIR` | `"results"` |
  | `public static final java.lang.String` | `RESULTS_MASTER_FILE` | `"MainTraits.csv"` |

- Package
- Class
- Tree
- Deprecated
- Index
- Help

*org.bioalg.riceprojecttype 1.0*

- Prev
- Next

- Frames
- No Frames

- All Classes
